# The Effects of Blunt Trauma and Cataract Surgery on Corneal Endothelial Cell Density

**DOI:** 10.4103/0974-9233.71604

**Published:** 2010

**Authors:** Baris Yeniad, Isik Corum, Cahit Ozgun

**Affiliations:** Department of Ophthalmology, Istanbul Faculty of Medicine, Istanbul University, Istanbul, Turkey

**Keywords:** Confocal Microscopy, Corneal Endothelium, Phacoemulsification, Senile Cataract, Traumatic Cataract

## Abstract

**Purpose:**

This study was designed to investigate the effects of trauma and cataract surgery on corneal endothelial cell density (ECD) in patients with a traumatic cataract due to blunt trauma without globe laceration.

**Materials and Methods:**

In this prospective study, 31 subjects with traumatic cataract (traumatic cataract group) and 30 subjects with a senile cataract (control group) were enrolled. The subjects with traumatic cataract were subdivided into two groups: uncomplicated surgery subgroup (*n* = 19) in which subjects underwent standard phacoemulsification with intraocular lens implantation and complicated surgery subgroup (*n* = 12) in which subjects underwent cataract surgery other than standard phacoemulsification. The ECD of the traumatic cataract group and the control group was compared preoperatively and at 3 months or later postoperatively. A P value less than 0.05 was considered statistically significant.

**Results:**

The ECD in the eyes with traumatic cataract was 13.1% lower than that for healthy eyes preoperatively (*P* = 0.043). Postsurgical ECD decreased by 16.7% in complicated surgery subgroup and 11.9% in uncomplicated surgery subgroup (*P* = 0.049) after 3 months postoperatively. The ECD decreased by 10.8% in the control group (*P* = 0.489).

**Conclusions:**

Patients with cataracts due to blunt trauma had a decreased endothelial cell count, which was significantly aggravated by cataract surgery. The loss of corneal endothelium cells due to surgery depends on the surgical approach.

## INTRODUCTION

The corneal endothelium plays an important role in maintaining corneal transparency. Corneal endothelial cell density (ECD) decreases with advanced age and intraocular surgery.^1^–^4^ Age and ECD show a negative correlation with endothelial cells decreasing in number by 0.1% for every year of life.^1^–^3^ Depending on the surgical approach during cataract surgery, up to 20% loss of endothelial cells has been reported.^5^–^7^

Lenticular injuries comprise 7% of ocular trauma, and they are the most common type of complication from penetrating ocular injuries that result in loss of vision.^8^,^9^ Traumatic cataracts are often associated with additional ocular complications that may affect the success of cataract surgery. The response of the corneal endothelium to ocular trauma may predict the results of surgery. The limited number of studies have focused on the impact of trauma on the corneal endothelium.^8^–^10^ Pong and Lai previously concluded that nonpenetrating ocular trauma causes a decrease in endothelial cell count. However, the difference was not significant compared to the control group.^10^ In another study, a decrease in mean endothelial cell count of 547 cells/mm^2^ was found in eyes with blunt ocular trauma.^4^

In this study, we investigate the effects of trauma and cataract surgery on ECD in patients with traumatic cataracts. We also compared the effects of complicated and uncomplicated surgery for traumatic cataracts.

## MATERIALS AND METHODS

**Figure 1:**
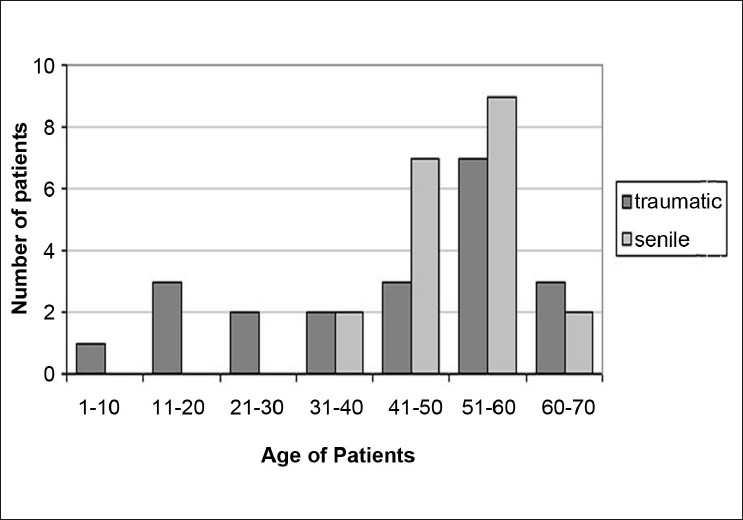
The age distribution of patients with traumatic and senile cataract

In this prospective study, 31 subjects with a traumatic cataract (traumatic cataract group) who presented to Department of Ophthalmology, Istanbul Faculty of Medicine, Istanbul University, between June 2005 and May 2007 were actively recruited. The control group comprised of 30 healthy subjects with senile cataract. The mean age of the subjects with traumatic cataracts was 48 years (range, 9–68 years) and 55 years (range, 48–72 years) for subjects with senile cataracts. The average age of subjects with traumatic cataracts was significantly lower than subjects with senile cataracts (*P* < 0.0001) [Figure 1].

Subjects with a recent history of blunt trauma without globe perforation with a healthy lens capsule in one eye and a healthy contralateral eye were included in the traumatic cataract group. The traumatic cataract group was subdivided into two groups based on the type of surgery: the uncomplicated surgery subgroup comprised 19 subjects who underwent standard phacoemulsification and intraocular lens implantation (Acrysof, Alcon Inc., Fort Worth, TX, USA) in the bag and with no peri-operative complications; the complicated surgery subgroup comprised 12 subjects who required additional surgical procedures for intraocular lens implantation. In the control group, 30 subjects with senile cataract who underwent uneventful phacoemulsification surgery and intraocular lens (Acrysof, Alcon Inc., Fort Worth, TX, USA). Subjects in the traumatic cataract group underwent surgery after resolution of symptoms such as eyelid edema, corneal edema, hyphema, and intraocular inflammation. All surgeries were performed by one surgeon (CO), and the AMO Sovereign device (Advanced Medical. Optics, Santa Ana, CA, USA) was used for all cataract surgeries.

In the complicated surgery subgroup, extracapsular cataract extraction (ECCE) was performed on five eyes, intracapsular cataract extraction (ICCE) was performed on three eyes, phacoemulsification was performed on two eyes, irrigation-aspiration without phacoemulsification was performed on one eye and the remaining eye developed zonular dialysis during phacoemulsification, and the lens nucleus dropped into the vitreous. The lens fragments in the vitreous were removed on a second surgery via pars plana vitrectomy. Additional procedures performed during surgery included corneal suturing in 12 eyes, anterior vitrectomy in 10 eyes, synechiolysis in one eye, and application of an iris retractor in one eye. Sulcus intraocular lens implantation was performed in five eyes, scleral fixated intraocular lens implantation in five eyes, in-the-bag intraocular lens implantation in one eye, and iris-claw intraocular lens in one eye.

Confocal microscopy was performed using the Confoscan 2.0 (NIDEK Co. Ltd., Gamagori, Japan) confocal microscope. NAVIS software (NIDEK Co. Ltd., Gamagori, Japan) was used for density measurements. Measurements were performed on the central cornea. Mean values were calculated with automatic analysis of three images that presented the best view of corneal endothelial cells. A region of interest (ROI) value of 0.0186 mm^2^ was used in all the measurements during analysis. Attention was paid to examine at least 0.0125 mm^2^ of valid area within this region. Erroneous evaluation of cell margins by the automated system were manually corrected. The procedure was repeated for both eyes of all subjects in the cohort, after the third postoperative month.

The Wilcoxon test was used to compare preoperative and postoperative values within a group. The Mann–Whitney *U*-test was used to compare the values between groups, whereas a Spearman correlation analysis was applied to assess relationship between parameters. SPSS 16.0 for Windows (SPSS Inc., Chicago, IL., USA) was used for statistical analyses, and *P* < 0.05 was statistically significant.

## RESULTS

**Figure 2:**
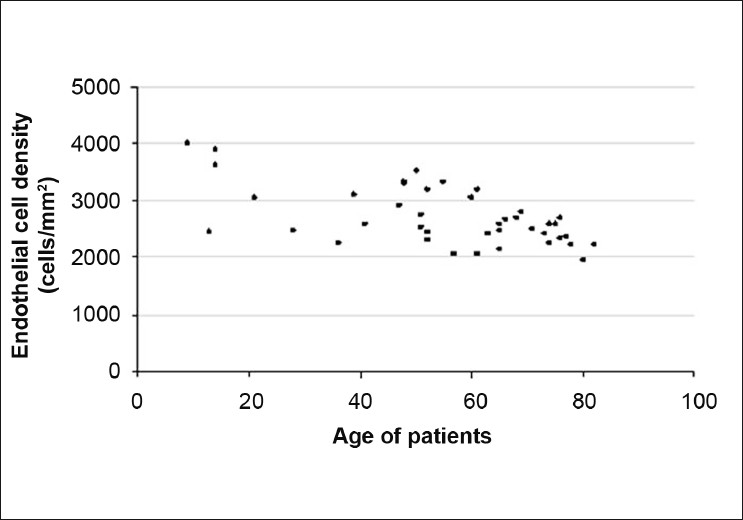
The distribution of endothelial cell density in healthy eyes

The distribution of ECD in 61 healthy eyes (30 eyes in the control group and 31 contralateral eyes in the traumatic cataract group) is shown in Figure 2. A statistically significant negative correlation was determined between age and ECD of healthy eyes (*P* < 0.0001, *r* = –0.527, Spearman correlation) [Figure 2].

The mean preoperative ECD in healthy eyes of traumatic cataract group was 2870 ± 558 cells/mm^2^, and 2493 ± 587 cells/mm^2^ in the traumatized eyes. ECD was 13.1% lower in traumatized eyes compared to healthy eyes preoperatively. The difference was statistically significant (*P* = 0.043). In the control group, no statistically significant difference was found between eyes (*P* = 0.829).

Table 1 shows the preoperative and postoperative mean ECD as well as the decrease in ECD in both groups. The mean decrease in ECD after 3 months postoperatively, was 333 ± 165 cells/mm^2^ in the traumatic cataract group and 276 ± 79 cells/mm^2^ the control group. This reduction in ECD in the traumatic cataract group was 13.3% and 10.8% in the control group. The decrease in ECD-associated surgery was statistically significant in both groups (*P* < 0.0001, both groups). However, no statistically significant difference was found between the two groups in terms of endothelial cell loss (*P* = 0.489).

**Table 1:** Preoperative and postoperative mean endothelial cell density and endothelial cell loss for the study groups

	Preoperative ECD, cells/mm^2^	Postoperative ECD, cells/mm^2^	Endothelial loss, cells/mm^2^	Endothelial loss, %	*P* value
Traumatic cataract group (*n* = 31)	2492.7 ± 587.0	2159.2 ± 612.2	333.5 ± 165.3	13.3	0.000
Control group (*n* = 30)	2582.6 ± 417.7	2306.5 ± 392.2	276.1 ± 78.8	10.8	0.000

ECD: Endothelial cell density, Traumatic cataract group comprised eyes that with a recent history of blunt trauma without globe perforation. Control group comprised eyes with senile cataract and no recent history of trauma. A *P* < 0.05 was considered statistically significant

**Figure 3:**
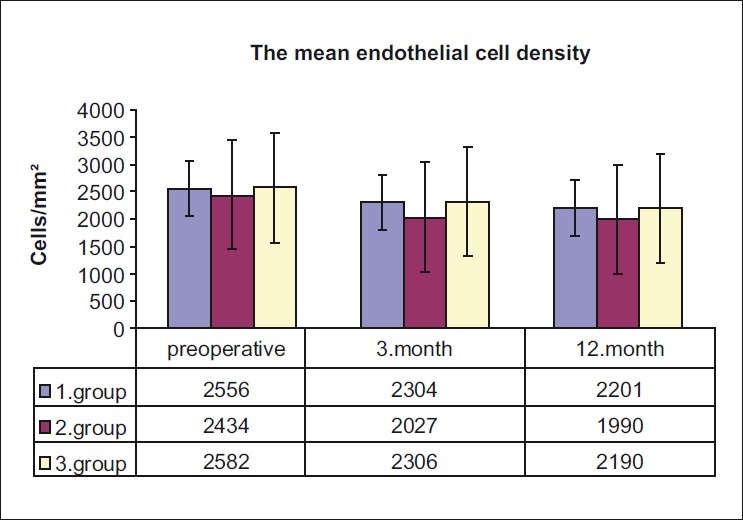
The mean endothelial cell density of three groups preoperatively and postoperatively at third and twelfth months. (1) Group: Uncomplicated surgery subgroup, (2) Group: Complicated surgery subgroup, and (3) Group: Control group

The preoperative ECD was not statistically different between the subgroups of the traumatic cataract group (*P* > 0.05). The level of endothelial cell loss associated with ECD and surgery in the subgroups is presented in Table 2. The mean endothelial cell loss was 407 ± 174 cells/mm^2^, in the complicated surgery subgroup and 293 ± 114 cells/mm^2^ in the uncomplicated surgery subgroup. These changes represented a 16.7% decrease in ECD in the complicated surgery subgroup and 11.9% in the uncomplicated surgery subgroup postoperatively. However, the degree of endothelial cell loss associated with surgery in the complicated cataract subgroup was found to be significantly higher (*P* = 0.049). The preoperative and postoperative mean endothelial cell densities are shown in Figure 3.

**Table 2:** The endothelial cell density in subjects with complicated or uncomplicated surgeries at the third postoperative month

	Uncomplicated surgery group	Complicated surgery group	*P* value
Preoperative ECD, cells/mm^2^	2556.6 ± 572.5	2434.7 ± 621.5	0.725
Endothelial loss, cells/mm^2^ (%)	-292.6 ± 114.5 (11.9%)	-407.1± 174.2 (16.7%)	0.049

ECD: Endothelial cell density, A *P* < 0.05 was considered statistically significant

## DISCUSSION

This study evaluated endothelial cell loss from cataract surgery in patients with traumatic cataract using confocal microscopy. Depending on the surgical approach, endothelial cell loss in cataract surgery may increase up to 40%.^5^,^8^,^11^ Many factors can influence ECD during cataract surgery, including technique, nucleus hardness, anterior chamber depth, phaco power and duration, irrigation volume, intracameral injections, irrigation fluids and viscoelastic agents, size and shape of the tunnel, type of intraocular lens implanted, postoperative inflammation, intraocular pressure, surgical experience, cataract type,^12^ and other factors.^13^–^16^ Recent advances in cataract surgery have focused on reducing surgical trauma, inflammatory response, and endothelial loss.^17^–^21^

In this study, while the ECD declined by a mean value of 13.3% in the traumatic cataract group following cataract surgery, it declined by 10.8% in the control group. Endothelial cell loss was found to be statistically significant for compared to preoperatively for both groups (*P* < 0.0001, both groups). However, no difference was identified between the traumatic cataract group and the control group in terms of endothelial cell loss (*P* = 0.489, respectively). The decrease in ECD was greater in the traumatic cataract group, but the difference between the two groups was not statistically significant. The outcome is likely due to the older age of the control group. In an age-matched study, Odenthal *et al*. implanted aphakic lenses in children with traumatic and congenital cataracts and found that the degree of ECD loss was significantly higher in eyes with previous trauma.^22^ Odenthal *et al*. concluded that the primary reason for endothelial cell loss was blunt ocular trauma rather than cataract surgery.^22^

There have been only a few studies that have focused on endothelial loss associated with ocular trauma. Slingsby *et al*. found that the traumatic eye had a mean decrease in ECD of 6.4% when compared to that in a normal eye.^23^ The decrease in ECD was correlated with the degree of trauma.^23^ Motley *et al*. analyzed ECD values in the eyes of pediatric patients who suffered from airbag-induced ocular trauma but did not undergo surgery and found a reduction of 547 cells/mm^2^.^4^ Sminia *et al*. reported a loss of 40% of endothelial cells in eyes with a traumatic cataract associated with perforating injury during the pediatric period.^8^ In another study, Odenthal *et al*. found 41% lower central ECD in the traumatic eyes compared to normal eyes.^22^ A significant negative linear correlation was found between the length of the corneal scar and the central corneal ECD.^22^

In this study, the ECD in traumatized eyes of subjects with a traumatic cataract was found to be 13.1% lower than that for the healthy eye (*P* = 0.043). In the control group, no statistically significant difference was found between the eyes of subjects with senile cataract and the healthy eyes (*P* = 0.829). This observation indicates that blunt ocular trauma had a negative effect on corneal ECD. During follow-up, the affected eyes in the control group (2582.6 ± 417.7 cells/mm^2^) and in the traumatic cataract group (2492.7 ± 587 cells/mm^2^) were compared in terms of ECD. No statistically significant difference was found (*P* = 0.434). This result was likely due to the older mean age in the control group.

In patients who have suffered blunt ocular trauma, a cataract may be the only clinical finding. Other manifestations may also influence the type of surgery, surgery time, complication rates, postoperative inflammation, and endothelial cell loss.^9^–^11^ Pandey *et al*. evaluated the results of capsular bag and ciliary sulcus fixation of IOL for traumatic cataracts and reported similar visual results, although fewer complications were associated with capsular bag fixation.^24^ Kodjikian *et al*. demonstrated an endothelial loss of 16% using pars plana phacofragmentation and Artisan lens implantation for traumatic subluxated cataracts at 12 months postoperatively.^25^

In the current study, 12 (38.7%) out of 31 subjects had one or more coexisting complications in addition to the cataract. The most common coexisting symptoms were iridodonesis (38%), phacodonesis (24%), and zonular rupture (24%). Ten (83%) out of 12 patients in the complicated surgery subgroup had additional symptoms, while only 3 (15.7%) out of the 19 patients in the uncomplicated surgery subgroup had additional symptoms. These findings verified the observation that additional symptoms make surgery more difficult and affect surgical outcomes. Hence, the ECD in the complicated surgery subgroup decreased by 16.7% following cataract surgery, whereas ECD in the uncomplicated surgery subgroup decreased by 11.9%. The preoperative ECD was not statistically significant different between the two subgroups; however, endothelial cell loss associated with surgery in the complicated surgery subgroup was statistically significantly higher (*P* = 0.049).

On the basis of our results and outcomes of previous studies,^26^ a certain level of corneal endothelial cell loss takes place due to cataract surgery. Endothelial cell loss can also occur as a result of trauma. Roper-Hall *et al*. concluded that trauma and intraocular surgery may have an additive effect on ECD in the cornea.^26^ In the current study, corneal endothelial cell loss in the traumatized eyes of cataract subjects without laceration was higher than that in the corresponding healthy eyes. While cataract may be the only finding in these patients, other findings may also be present. However, in the presence of coexisting symptoms, cataract surgery becomes more complicated and more corneal endothelial cell loss is observed depending on the complexity of the surgical approach.
